# Chitooligosaccharides Improve the Efficacy of Checkpoint Inhibitors in a Mouse Model of Lung Cancer

**DOI:** 10.3390/pharmaceutics14051046

**Published:** 2022-05-12

**Authors:** Astrid Zedlitz Johansen, Marco Carretta, Marie-Louise Thorseth, Shawez Khan, Klaire Yixin Fjæstad, Christian Beltoft Brøchner, Hannes Linder, Christina Ankjærgaard, Marco Donia, Inna Chen, Dorte Lisbet Nielsen, Claus Preibisch Behrens, Daniel Hargbøl Madsen

**Affiliations:** 1 National Center for Cancer Immune Therapy, Department of Oncology, Copenhagen University Hospital—Herlev and Gentofte, 2730 Herlev, Denmark; marco.carretta@regionh.dk (M.C.); marie-louise.thorseth@regionh.dk (M.-L.T.); shawez.khan@regionh.dk (S.K.); klaire.yixin.fjaestad@regionh.dk (K.Y.F.); hannes.linder@regionh.dk (H.L.); marco.donia@regionh.dk (M.D.); daniel.hargboel.madsen@regionh.dk (D.H.M.); 2 Department of Pathology, Centre of Diagnostic Investigations, Copenhagen University Hospital—Rigshospitalet, 2100 Copenhagen, Denmark; christian.beltoft.broechner@regionh.dk; 3 Department of Health Technology, Technical University of Denmark, 4000 Roskilde, Denmark; cank@dtu.dk (C.A.); claus.behrens@regionh.dk (C.P.B.); 4 Department of Oncology, Copenhagen University Hospital—Herlev and Gentofte, 2730 Herlev, Denmark; inna.chen@regionh.dk (I.C.); dorte.nielsen.01@regionh.dk (D.L.N.); 5 Department of Clinical Medicine, Faculty of Health and Medical Sciences, University of Copenhagen, 2200 Copenhagen, Denmark; 6 Department of Immunology and Microbiology, Faculty of Health and Medical Sciences, University of Copenhagen, 2200 Copenhagen, Denmark

**Keywords:** checkpoint inhibitors, chitin, chitooligosaccharides, immunotherapy, lung cancer, radiotherapy, syngeneic mouse cancer models, YKL-40

## Abstract

YKL-40 (also named chitinase 3 like-1 protein [CHI3L1]) is a secreted chitinase-like protein which is upregulated in cancers and suggested to have pro-tumorigenic activity. YKL-40 lacks enzymatic function, but it can bind carbohydrates such as chitin. Chitooligosaccharides (COS) derived from deacetylation and hydrolysis of chitin might be used for the blockade of YKL-40 function. Here, public single-cell RNA sequencing datasets were used to elucidate the cellular source of YKL-40 gene expression in human tumors. Fibroblasts and myeloid cells were the primary sources of YKL-40. Screening of YKL-40 gene expression in syngeneic mouse cancer models showed the highest expression in the Lewis lung carcinoma (LL2) model. LL2 was used to investigate COS monotherapy and combinations with immune checkpoint inhibitors (anti-PD-L1 and anti-CTLA-4) (ICIs) and radiotherapy (8 Gy × 3) (RT). COS tended to reduce plasma YKL-40 levels, but it did not affect tumor growth. LL2 showed minimal responses to ICIs, or to RT alone. Interestingly, ICIs combined with COS led to delayed tumor growth. RT also enhanced the efficacy of ICIs; however, the addition of COS did not further delay the tumor growth. COS may exert their anti-tumorigenic effects through the inhibition of YKL-40, but additional functions of COS should be investigated.

## 1. Introduction

Chitin is a highly abundant glycopolymer. It is an essential component of fungal cell walls, crustacean shells, insect exoskeletons, and some parasites. Deacetylation and hydrolysis of chitin result in the formation of chitosan [1]. Chitosan is not as prevalent as chitin, although specific bacteria and fungi species secrete chitin deacetylases that catalyze the conversion of chitin to chitosan [2]. Chitin and chitosan are non-toxic, biodegradable, and biocompatible compounds. Chitosan can be hydrolyzed to chitooligosaccharides (COS) with a more uniform molecular size, lower molecular weight, and improved solubility in neutral pH solvents. The most common biomedical applications of chitosan and COS are their use as adjuvants, in particle or gel form, or as drug delivery systems for metabolic or anti-neoplastic agents [3,4]. They are also used as tissue scaffolds and wound dressings [5]. Chitosan and COS show favorable immunomodulatory, anti-oxidant, anti-microbial, and anti-tumor properties [5].

YKL-40 (also known as chitinase-3-like 1 [CHI3L1]) is a conserved, secreted chitinase-like protein (CLP) found in elevated levels in many different diseases characterized by inflammation and extracellular remodeling, including cancer [6,7]. Elevated plasma YKL-40 is associated with poor survival in patients with various solid cancer types [8]. CLPs lack the enzymatic function required for catalyzing the hydrolysis of chitin, but they have retained their chitin-binding properties [9]. YKL-40 is the most studied CLP, and it is suggested as a therapeutic target in different diseases, including cancer as well as inflammatory and neurological diseases [10]. YKL-40 can bind chitin, heparin, collagen, and hyaluronan, and it has been suggested to promote cancer through binding to the IL-13Rα2, RAGE, syndecan-1, and CD44v3 receptors on the surface of cancer cells [7].

Chitin, chitosan, and COS have been investigated for their anti-tumor properties. In vitro, administration leads to reduced matrix metalloproteinases expression, increased apoptosis, and decreased proliferation of cancer cells [11,12,13,14,15,16,17]. Little is known about the potential immune modulatory effects of these compounds, although in vitro administration has been shown to increase the cytotoxicity of lymphocytes [18,19]. In vivo, oral or intraperitoneal administration of chitosan and COS has shown anti-tumor activity in mouse cancer models of sarcoma [18,20], melanoma [19], and Ehrlich-Lettre ascites carcinoma [11] as well as liver [13], lung [13,21], colorectal [22], and kidney cancer [16]. Increased apoptosis [11,16], inhibition of angiogenesis [11], and beneficial changes in the expression of genes involved in NF-κB- and mTOR-signaling as well as dendritic and natural killer cell responses [19,22] were observed after treatment with chitosan or COS.

Few studies have examined YKL-40 expression after treatment with chitin or COS, but the administration of COS to cultured macrophages has been shown to reduce YKL-40 secretion [23]. Furthermore, treatment with chitin microparticles reduced plasma YKL-40 levels in a mouse breast cancer model [24]. In addition to the direct blocking of YKL-40, an alternative mechanism underlying the observed anti-tumor effects could therefore involve a COS-mediated reduction in YKL-40 levels. 

This study aimed to investigate the gene expression of YKL-40 in specific cell populations present in healthy and cancer tissue, and the overall expression in different syngeneic mouse cancer models. The effect of targeting YKL-40 with COS in combination with immune checkpoint inhibitors (ICIs) and radiotherapy (RT) was examined. 

## 2. Materials and Methods

### 2.1. Single-Cell RNA Sequencing Datasets from Public Repositories

Raw single-cell RNA sequencing (scRNAseq) data were downloaded from public repositories after surveying the literature. Raw scRNAseq data and cell type annotations from lung, colorectal, and ovarian cancer were downloaded from EMBL-EBI accession number E-MTAB-8107 [25]. To investigate similarities across datasets, raw scRNAseq data on lung and colorectal cancer and cell type annotations were downloaded from Gene Expression Omnibus (GEO) using accession numbers GSE131907 [26] and GSE166555 [27], respectively. Data from cancer tissue samples without matched, adjacent healthy tissue samples were removed. Raw data were processed further in R (version 4.0.1). The following quality control steps were performed: (i) genes expressed by less than 3 cells were not considered; (ii) cells that expressed fewer than 200 genes (low quality) were excluded. The data were normalized using the *SCTransform* function as implemented in the Seurat R package [28]. Data quantification for the figures was performed using the open-source software tool BIOMEX (version 1.0-1) [29].

### 2.2. Cell Lines

The B16F10 melanoma, Lewis lung (LL2), MC38 colon, and PANO2 pancreatic cancer cell lines were cultured in complete growth media of DMEM (cat. no. 11995-065, Gibco, Waltham, MA, USA) supplemented with 10% FBS (cat. no. 10500-064, Gibco) and 1% P/S (cat. no. 15140-122, Gibco). The EO771.LMB breast cancer cell line was cultured in DMEM supplemented with 20% FBS, 1% P/S, and 20 mM HEPES (cat. no. 15630-056, Gibco).

### 2.3. In Vivo Experiments

Animal experiments were conducted at the animal facility of the Department of Oncology, Copenhagen University Hospital—Herlev and Gentofte, Herlev, Denmark, following ARRIVE guidelines and with approval from the Danish Animal Experiments Inspectorate. Daily care was performed by animal caretakers. Female 8–17-week-old C57BL/6 mice (Taconic) were housed under standard conditions. For all tumor models, a total of 5 × 10^5^ cancer cells in 100 µL phosphate-buffered saline (cat. no. D8537, Sigma-Aldrich, St. Louis, MO, USA) (PBS) were subcutaneously injected into the right flank. The tumor growth was evaluated by measuring tumor dimensions (length and width) three times weekly using a digital caliper. The tumor volume was calculated as (width^2^ × length) × (π/6). For comparison of the different tumor models, tumors were harvested at similar sizes, which, depending on the model, required between 14 and 40 days of growth in vivo. For survival studies, the mice were sacrificed when tumor size reached >1000 mm^3^ or at a humane endpoint defined as either wound on tumors of more than 6 mm, tumor collapse, weight loss >20%, or general distress. The blood and tumors were collected for further analysis. 

### 2.4. Therapies

COS were kindly provided by Genis (T-ChOS, Genis, Siglufjordur, Iceland). The powder was produced from deacetylation of chitin flakes from shrimp shells and characterized to be 60% deacetylated, with 78.5% of the powder having a degree of polymerization at 2–6 [23]. A fresh stock of COS was dissolved in sterile PBS before each study and stored at 4 °C for no more than 2 months. The InVivoMAb anti-mouse-PD-L1 (clone 10F.9G2, cat. no. BE0101) and anti-mouse-CTLA-4 (clone 9D9, cat. no. BE0164) were from BioXcell. Doses of 1 mg COS/mouse and 200 µg of each antibody/mouse were diluted in 200 µL sterile PBS prior to treatment and injected intraperitoneally. The chosen dose of COS was based on other studies showing anti-tumor effects in mouse models at this dose [21,24]. Treatment was initiated 2 days after inoculation of cancer cells and continued every 3rd day until an endpoint was reached. For the study involving the combination with RT, treatment was initiated 5 days after cancer cell inoculation. Control mice were treated with PBS. 

### 2.5. Radiotherapy

RT was provided following a newly described method for treating subcutaneous mouse flank tumors using a clinical linear accelerator [30]. Briefly, individual mice were transferred to an induction chamber and anesthetized using air with 4% isoflurane (Univentor 40 Anestheasia Unit, AgnThos, Lidingö, Sweden). The individual mice were positioned on the left flank, anesthetized through a face mask using air with 2.8% isoflurane, and irradiated with a planned dose of 8 Gy from a 3 × 3 cm^2^ flattening filter free 10 megavoltage beam (TrueBeam treatment unit, Varian Medical Systems, Palo Alto, CA, USA). Care was taken to avoid normal tissue and the field was slightly shaped by the multi-leaf collimator. Three tumor-free mice of similar weight were placed in the same position, and alanine pellets (batch DC600, Harwell Dosimeters, Didcot, UK) were used to validate the planned dose. The pellets were placed on top of the skin mimicking tumor placement, and temperature was measured prior to RT. The stable free radicals induced in the pellets during RT were measured using electron paramagnetic spin resonance [30].

### 2.6. Gene Expression Analysis

Cell pellets from cultured cancer cells and fragments from excised tumors were stored in RNAlater (cat. no. AM7020, InVitrogen, Waltham, MA, USA). RNA was extracted with the RNeasy Mini Kit (cat. no. 74104, Qiagen, Hilden, Germany) using the manufacturer’s instructions. The RNA concentration and quality were measured using a NanoDrop spectrophotometer (Thermo Scientific, Waltham, MA, USA). RNA was reverse-transcribed into cDNA using the iScript cDNA Synthesis Kit (cat. no. 1708891, Biorad, Hercules, CA, USA) according to the manufacturer’s instructions. The quantitative real-time PCR (qRT-PCR) was performed using the Brilliant III Ultra-Fast SYBR^®^ dye system (cat. no. 600882, Agilent, Santa Clara, CA, USA) with ROX as a reference dye. The reactions were run on an AriaMx Real-Time PCR system (Agilent). The thermal profile was 1 cycle at 95 °C for 3 min and 40 cycles of 95 °C for 5 s; 60 °C for 20 s followed by a melting curve analysis of 65–95 °C with 0.5 °C increment; 5 s per step. All samples were run in triplicates. The data were normalized to the expression level of the housekeeping gene β-actin. Data were analyzed with the 2^−ΔΔCt^ method, and fold changes of the individual samples were compared to the sample with the lowest expression. Primer sequences were: YKL-40, 5′ forward 3′: CAGATAGCCCACACCTGGAT and 5′ reverse 3′: GTGATGGCCTGTGATTTGGC; β-actin, 5′ forward 3′: CACTGTCGAGTCGCGTCC and 5′ reverse 3′: TCATCCATGGCGAACTGGTG. Specific amplification was evaluated with no-reverse transcriptase controls (cDNA reactions without reverse transcriptase) and no-template controls (reactions without cDNA). Whole-tumor RNA sequencing data on fragments from excised tumors were available from Carretta and Madsen et al. (manuscription in preparation). 

### 2.7. Immunohistochemistry

Excised tumor pieces were fixed in 4% formaldehyde overnight at 4 °C and transferred to 70% ethanol for continuous storing at 4 °C. Tissues were embedded in paraffin, cut into 1.5 μm tissue sections (Microm HM355S, Thermo Scientific), and placed on silanized glass slides. For hematoxylin and eosin staining, the sections were placed for 10 min in Mayers hematoxylin (cat. no. AMPQ00253.5000, VWR, Radnor, PA, USA), followed by 10 min in running water, and 2 min in eosin (cat. no. 115935, Sigma-Aldrich). For YKL-40 staining, endogenous peroxidase was quenched using a 0.5% solution of hydrogen peroxide in TRIS-buffered saline for 15 min, prior to overnight incubation with an anti-YKL-40 antibody (1:400, cat. no. 12036-1-AP, Proteintech, Rosemont, IL, USA) at 4 °C, diluted in blocking buffer. The Dako REAL EnVision Detection System (cat. no. K5007, Agilent) was used for detecting the primary antibody The sections were washed with TBS, followed by incubation for 10 min with a 3,3′-diamino-benzidine chromogen solution. Positive staining was recognized as a brown color. The sections were counterstained with Mayers hematoxylin, dehydrated in graded alcohols, and coverslipped with Pertex mounting medium (cat. no. 00801, HistoLab, Askim, Sweden). The tissue sections were evaluated by a consultant surgical pathologist. Staining was assessed in tumor and stroma cells using a modified Allred scoring based on the sum of a proportion and an intensity score. The Allred scoring was converted to a scale from ‘−’ = no expression to ‘+’ = low expression, ‘++’ = moderate expression, and ‘+++’ = high expression. 

### 2.8. Multicolor Flow Cytometry

Excised LL2 tumors were cut into small pieces and enzymatically digested overnight with 2.1 mg/mL collagenase type I (cat. no. LS004196, Worthington, Columbus, OH, USA), 75 µg/mL DNase I (cat. no. LS002139, Worthington), and 5 mM CaCl_2_ in RPMI (cat. no. 72400-021, Gibco) using an end-over-end rotor at 4 °C. The next day, tumors were incubated in a thermal shaker at 37 °C for 15 min, at 300 rpm. The digested tumors were forced through a 70 µm filter to obtain a single-cell suspension. Red blood cells were lyzed with RBC lysis buffer (cat. No. 1045722, Qiagen) for 5 min. Cells were incubated with FcR block (cat. No. 1309-092-575, Miltenyi, Bergisch Gladbach, Germany) at 4 °C for 10 min and stained with CD45-FITC (cat. No. 103107, BioLegend, San Diego, CA, USA), PDGFRα-BV605 (cat. No. 135916, BioLegend), CD4-BV421 (cat. No. 100438, BioLegend), CD8-PerCP-Cy5.5 (cat. No. 551162, BD Pharmingen, San Jose, CA, USA), CD11b-PE/Cy7 (cat. No. 101216, BioLegend), F4/80-APC (cat. No. 123,116 BioLegend), MMR-PE (cat. No. 141706, BioLegend), and a biotinylated FAP antibody (cat. no. BAF3715, R&D Systems, Minneapolis, MN, USA) at 4 °C in the dark for 20 min. After washing, cells were stained with Streptavidin-APC/Cy7 (cat. no. 405208, BioLegend) at 4 °C in the dark for 10 min. Viability was assessed with Zombie Aqua Fixable Viability Kit (cat. no. 423102, BioLegend). Data were acquired on an ACEA Quanteon flow cytometer and analyzed with Novo Express software (Agilent). The gating strategy is found in Supplementary Figure S1. 

### 2.9. ELISA

Blood was collected from mice at the time of sacrifice and coagulated at room temperature for 30 min. Samples were centrifuged for 10 min at 1500× *g* and 4 °C, and serum was transferred to a 1.5 mL Eppendorf tube and stored at −80 °C. Mouse YKL-40 was measured using the mouse YKL-40 quantikine ELISA kit (cat. no. MC3L10, R&D Systems) using the manufacturer’s instructions. The samples were diluted 1:75 and run in duplicates. 

### 2.10. Statistical Analysis

Statistical analyses were performed with Prism 8 (GraphPad, San Diego, CA, USA), except for comparison of tumor growth curves. Mann–Whitney U tests were used to test if two samples were likely to originate from similar distributions. The open-access software TumGrowth [31] was used to compare tumor growth curves with linear mixed-effect modelling using the default settings. Mice sacrificed according to a humane endpoint before reaching the defined maximum tumor size of 1000 mm^3^ were excluded from the survival analysis using the log-rank test. A *p* < 0.05 was considered significant.

## 3. Results

### 3.1. Fibroblasts and Myeloid Cell Populations Are the Main Producers of YKL-40

**Figure 1:**
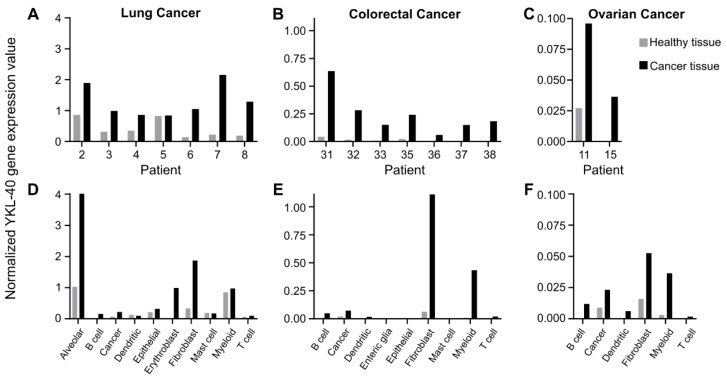
ScRNAseq data from patients with lung, colorectal, and ovarian cancer. Seurat-based normalized YKL-40 gene expression value comparing healthy tissue with cancer tissue in (**A**–**C**) individual patients and in (**D**–**F**) cell subsets for: lung (*n* = 7), colorectal (*n* = 7), and ovarian cancer (*n* = 2) (E-MTAB-8107). For gene annotation of cell populations, please refer to Qian et al. [25].

Public scRNAseq data were used to investigate which cell type(s) is responsible for the elevated YKL-40 expression found in cancer tissues from patients with early-stage lung cancer, mixed-stage colorectal cancer, and ovarian cancer. For all patients, YKL-40 gene expression was higher in biopsies from cancer tissue compared to adjacent, matched healthy tissue (Figure 1A–C). There was, in general, low or no detectable YKL-40 expression in the healthy tissue. YKL-40 gene expression was higher in patients with lung cancer compared to colorectal cancer (Figure 1A,B). Low expression values were observed for ovarian cancer (Figure 1C). YKL-40 gene expression was primarily increased in the fibroblast (gene annotation: *COL1A*, *BGN*, *DCN*), myeloid (gene annotation: *CD68*, *LYZ*, *AIF1*), cancer (gene annotation: *EPCAM*, *KRT7*, *KRT18*), and B-cell (gene annotation: *CD79A*, *CD79B*) populations in cancer tissue compared to healthy tissue (Figure 1D–F). Specifically for lung cancer, the highest expression level was observed in the alveolar (gene annotation: *CLDN18*, *SFTPA1*, *SFTPA2*, *SFTPC*) cell population, and erythroblasts (gene annotation not available) also increased their expression (Figure 1D). 

Similar results were observed using two other scRNAseq datasets for early-stage lung cancer and mixed-stage colorectal cancer (Supplementary Figure S2). Overall, the analysis indicated that the upregulation of YKL-40 in fibroblasts and myeloid cells is the main reason for increased YKL-40 expression in cancer tissues.

### 3.2. YKL-40 Gene and Protein Expression in Syngeneic Mouse Cancer Models

**Figure 2:**
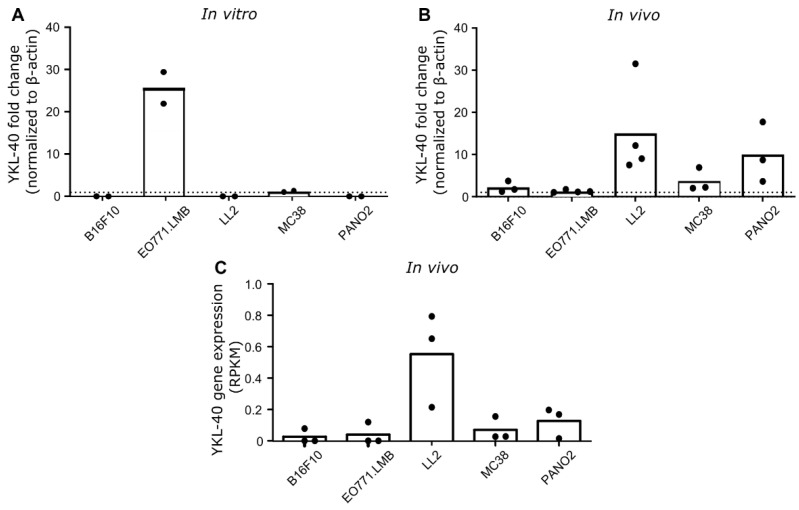
YKL-40 gene expression in mouse cancer cell lines and engrafted tumors. RNA was isolated from (**A**) cultured cell lines (*n* = 2 per group) and (**B**) engrafted tumors (*n* = 3–4 per group), and YKL-40 expression levels were analyzed with RT-qPCR. Samples were run in technical triplicates, and expression levels were normalized to the β-actin housekeeping gene and illustrated as fold change compared to the cell line with the lowest expression level (mean). Dotted line at y = 1. (**C**) YKL-40 gene expression after sequencing of RNA isolated from engrafted tumors (*n* = 3 per group) (mean). Abbreviations: RPKM, reads per kilobase million.

YKL-40 gene expression was investigated in five different cultured mouse cancer cell lines and in engrafted tumors derived from these cell lines. Based on qRT-PCR analysis of cultured cells, the highest level of YKL-40 gene expression was detected in EO771.LMB breast cancer cells, whereas low levels were observed in MC38 colon cancer and undetectable levels in B16F10 melanoma cells, LL2 lung cancer cells, and PANO2 pancreatic cancer cells (Figure 2A). YKL-40 gene expression was also measured using qRT-PCR analysis of whole-tumor RNA isolated from tumor fragments. Surprisingly, the highest levels were observed in LL2 tumors, followed by PANO2 and MC38. Low levels were observed in B16F10 and EO771.LMB (Figure 2B). A very similar YKL-40 gene expression pattern was observed based on RNA sequencing of the same tumor models (Figure 2C). 

Tumor tissue sections from the different mouse models were immunostained for investigation of YKL-40 protein expression. Expression was observed in both cancer and stromal cells (Table 1). It should, however, be noted that cancer cells were predominantly observed as negative (Supplementary Figure S3), and that positive staining in cancer cells close to necrotic areas contributed to the relatively high score in this cell population. In general, comparable scorings were observed in the stroma of the different tumor models, except for EO771.LMB, where the lowest scoring was observed (Table 1). These observations indicate that stromal cells are important contributors of YKL-40 in mouse tumors.

### 3.3. COS Improve the Effect of ICIs in the LL2 Model

**Figure 3:**
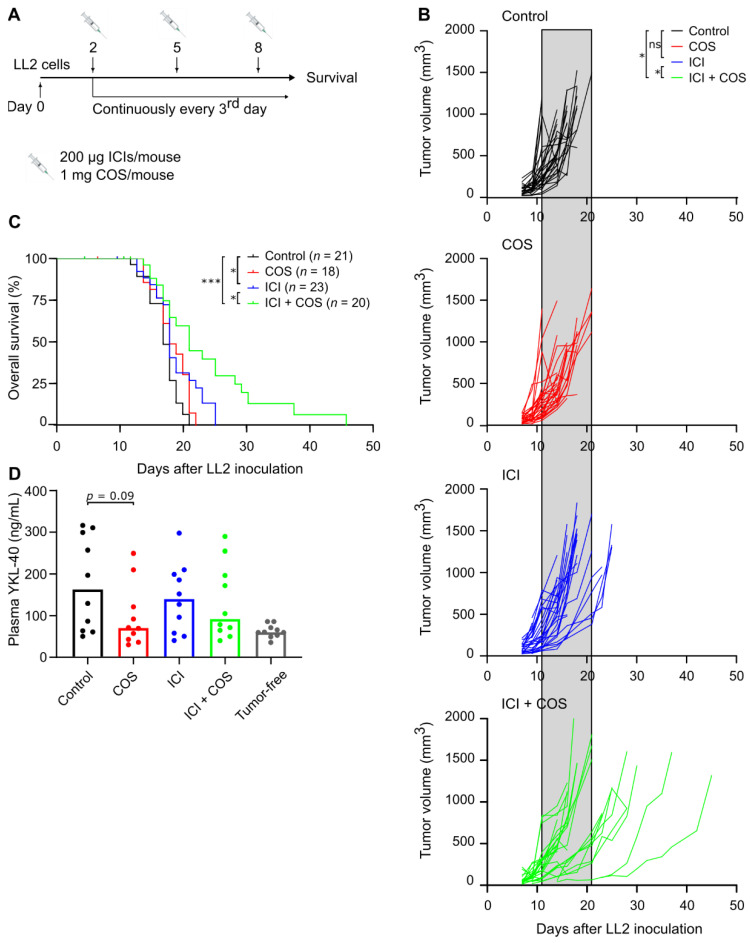
COS improve the efficacy of an ICI cocktail, anti-PD-L1 and anti-CTLA-4, in the LL2 lung cancer model. (**A**) Treatment regimen and experimental setup of tumor study. (**B**) Individual tumor growth curves for C57BL/6 mice inoculated with 5 × 10^5^ LL2 cancer cells (*n* = 30 per group). Shaded areas included for easy comparison of the different groups represent the terminal tumor growth time frame of the control group. (**C**) Kaplan–Meier plot of survival curves. (**D**) Plasma YKL-40 levels at time of sacrifice (*n* = 10 per group) (mean). * *p* < 0.05, *** *p* < 0.001, according to Mann–Whitney U test. Abbreviations: COS: chitooligosaccharides; ICI: immune checkpoint inhibitors; ns: not significant.

LL2 was selected for investigation of COS as monotherapy or in combination with ICIs due to the highest YKL-40 gene expression among the tumor models. Mice subcutaneously inoculated with LL2 cancer cells were treated with COS or an ICI cocktail consisting of anti-PD-L1 and anti-CTLA-4 antibodies (Figure 3A). COS alone did not affect the tumor growth or survival of the mice (Figure 3B,C). Treatment with ICIs resulted in reduced tumor growth in individual mice (Figure 3B) and prolonged survival compared to control mice (Figure 3C). Strikingly, the combination of COS and ICIs led to delayed tumor growth and prolonged survival compared to the single treatments (Figure 3B,C). A trend towards lower plasma YKL-40 in the treatment groups that received COS was observed. These levels were comparable to plasma YKL-40 levels in tumor-free C57BL/6 mice (Figure 3D). 

To investigate potential effects of COS and ICI on the cellular composition of the tumor microenvironment, LL2 tumors were analyzed by multicolor flow cytometry. No clear differences were observed, but there was a trend towards higher percentages of CD4+ and CD8+ cells after treatment with ICIs with/without COS (Supplementary Figure S4). The LL2 model is known as a very aggressive tumor model that responds poorly to ICIs [32,33,34,35]. To test if COS could have an anti-tumor effect as a monotherapy in a more immunogenic model, mice were subcutaneously inoculated with MC38 cancer cells and treated with COS. There was no difference in tumor growth between control mice and COS-treated mice (Supplementary Figure S5). 

### 3.4. Combination of ICIs, RT, and COS in the LL2 Model

**Figure 4:**
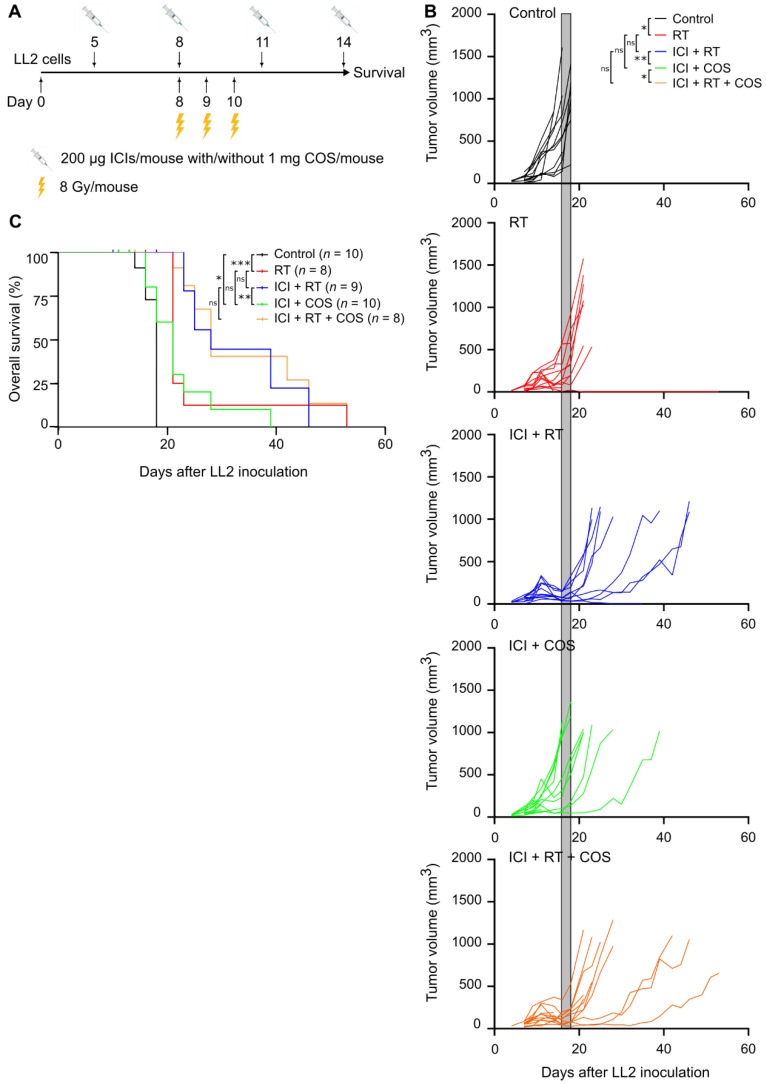
Targeting YKL-40 with COS in combination with ICIs and RT in the LL2 model. (**A**) Treatment regimen and experimental setup of tumor study. (**B**) Individual tumor growth curves for C57BL/6 mice inoculated with 5 × 10^5^ LL2 cancer cells (*n* = 12 per group). Shaded areas included for easy comparison of the different groups represent the terminal tumor growth time frame of the control group. (**C**) Kaplan–Meier plot of survival curves. * *p* < 0.05, ** *p* < 0.01, *** *p* < 0.001. Abbreviations: COS: chitooligosaccharides; ICI: immune checkpoint inhibitors; ns: not significant; RT: radiotherapy.

A total of 55% (11 out of 20) of LL2 tumors did not respond to treatment with ICIs combined with COS (Figure 3B,C). Therefore, the effect of adding localized, fractionated RT was investigated (Figure 4A). The RT dose was validated daily during the three-day treatment period using alanine pellets as dosimeters. The dose of the individual alanine pellets ranged from (7.69 ± 0.36) Gy to (8.01 ± 0.36) Gy, verifying the planned dose of 8 Gy (data not shown). RT as monotherapy led to a small reduction in tumor growth and prolonged survival (Figure 4B,C). The combination of COS and ICI also delayed tumor growth and increased survival compared to control mice (Figure 4B,C). Interestingly, the combination of RT and ICIs appeared particularly potent and led to delayed tumor growth and prolonged survival compared to mice treated with RT as monotherapy (Figure 4B,C). The addition of COS did not further improve this treatment (Figure 4B,C).

## 4. Discussion

YKL-40 is a prognostic biomarker in many solid cancers, and elevated plasma YKL-40 levels are associated with poor survival [8]. Studies have shown anti-tumor effects of YKL-40 blockade using antibodies or natural compounds such as chitin [24,36]. It is not fully elucidated which cell type(s) contributes to elevated YKL-40 in cancer, and it has not been investigated if YKL-40 blockade could improve the efficacy of ICIs and RT. In this study, we investigated the cellular sources of YKL-40 gene expression in the tumor microenvironment using public scRNAseq datasets. Additionally, we used syngeneic mouse cancer models to study the potential anti-tumor effect of the YKL-40 binding compound COS as a monotherapy or combined with ICIs and RT. 

Previous studies have investigated YKL-40 gene expression in whole tumors [37,38,39]. The availability of public scRNAseq data allowed us to investigate the expression in specific cell populations of the tumor microenvironment. We found that, primarily, fibroblast and myeloid cell populations (including alveolar macrophages in patients with lung cancer) increase their expression of YKL-40, and that cancer cells do so to a lesser extent. This suggests that fibroblasts and myeloid cells are the primary producers of YKL-40 in the tumor microenvironment. 

We investigated YKL-40 expression in cultured cell lines and in engrafted tumors derived from five common mouse cancer cell lines of different cancer types originating from the C57BL/6 mouse strain. YKL-40 was detected in cultured EO771.LMB and MC38, but not in cultured B16F10, LL2, and PANO2. The highest expression of YKL-40 was found in LL2 tumors, whereas B16F10 and EO771.LMB had the lowest expression, and YKL-40 was only detected in 1 out of 3 tumors after RNA sequencing. Others have shown a similar absence in cultured human cancer cell lines and an up-regulation of YKL-40 in xenograft tumors [40]. One explanation for this could be that YKL-40 is mainly expressed by other cells than cancer cells, as also indicated by our analysis of scRNAseq data and immunohistochemistry. Another explanation could involve the different environments that cells experience in vivo compared to in vitro, which could stimulate upregulation of YKL-40 in cancer cells. An elucidation of YKL-40 expression is important for model selection in future studies investigating the role of YKL-40 in cancer. Overall, our data show that YKL-40 expression in cultured cancer cells does not necessarily reflect the expression levels in the formed tumors, as observed by others [40]. 

Among the tested tumor models, the highest YKL-40 expression was found in LL2 tumors. This model was therefore used to study the potential anti-tumor effect of COS. Previous studies have shown that COS administration leads to reduced tumor growth and metastatic spread in LL2-tumor-bearing mice [13,21]. In our study, however, we did not observe any effect of COS on tumor growth. It should be noted that the different COS preparations used in this and other pre-clinical studies could influence the obtained results. Chitin can be isolated from numerous natural sources, for example, fungi and crustaceans, and can be processed in different ways. Furthermore, COS are a family of carbohydrates that differ due to the method of preparation, degree of deacetylation, molecular weight, size, and purity. It has previously been shown that the molecular size of COS influenced the anti-tumor effect in a sarcoma model [18]. 

In most studies, LL2 tumors are reported as non-responsive to ICIs [32,34,35] although examples of LLC tumors responding to ICIs also exist [41]. Combinations of anti-PD-1/PD-L1 and anti-CTLA-4 may have complementary action, resulting in better tumor response [42]. Here, the ICI treatment, consisting of anti-CTLA-4 and anti-PD-L1, appeared to delay tumor growth in some of the mice, whereas many tumors showed primary resistance to the treatment. A combination of ICIs with treatment that increases immunogenicity of the cancer cells or modulates the immune environment has been shown to enhance the response to ICIs [43,44]. Interestingly, we found that the addition of COS to ICIs delayed tumor growth and prolonged survival compared to monotherapies. This is in alignment with a previous study, which demonstrated a synergistic effect of combining antibody-based blocking of YKL-40 with ICIs in the B16F10 tumor model [36]. LL2 is a fast-growing and aggressive model that does not respond well to ICIs, whereas MC38 is more immunogenic and more likely to respond to immunotherapy [32,33]. Therefore, the immunomodulatory effects of COS could potentially lead to an anti-tumor effect in this tumor model, even as a monotherapy. However, COS did not result in any observable effect on tumor growth in the MC38 model either. One reason for this could be the lower expression level of YKL-40 in the MC38 model compared to the LL2 model. The observation that COS as monotherapy did not delay tumor growth but that it potentiated the effect of ICIs suggests that the effect of COS may involve other cells of the tumor microenvironment. Using flow cytometry, we investigated if COS treatment as monotherapy or in combination with ICIs led to changes in the cellular composition of the tumors. We did not observe any differences that could explain the anti-tumor effect of COS, but we did see a trend towards higher percentages of CD4+ and CD8+ cells after treatment with ICIs. Further efforts to elucidate the mechanism of action of COS should involve studies of the effects on the phenotype of specific cell types and on signaling pathways activated by COS treatment. 

YKL-40 can bind to different receptors expressed on different cell types. These include IL13Rα2, RAGE, syndecan-1, and CD44v3. Signaling through these receptors can initiate cancer-promoting signaling and inhibit cytotoxic lymphocytes [7]. It is unknown which YKL-40-receptor binding is the one(s) leading to cancer-promoting effects observed in this and other pre-clinical studies. Recently, the chitin-binding domain was associated with the binding domain to IL13Rα2, suggesting that COS could block the interaction between YKL-40 and IL13Rα2 [45]. This could be one of the ways that COS exert their effects on tumor growth. Evidence that directly links the effects of COS with YKL-40 blockade is still lacking. In addition to directly blocking YKL-40, COS treatment could also lead to reduced plasma YKL-40 levels. Reduced plasma YKL-40 was observed after the administration of chitin microparticles [24]. However, it cannot be ruled out that this could be due to steric hindrance of antibody binding during the ELISA-based detection leading to an underestimation of the YKL-40 levels observed after COS treatment.

Chitosan and COS have also been suggested to promote innate and adaptive immune response by promoting the cGAS-STING-dependent induction of type 1 interferons in dendritic cells [46], the inhibition of PI3K-AKT signaling involved in cancer proliferation and survival [47], and the induction of apoptosis [48]. These functions of COS, which are independent of YKL-40, could also lead to the observed anti-tumor effect. 

As many individual tumors remained resistant to treatment with ICIs combined with COS, RT was added. LL2 tumors were largely resistant to RT delivered as 8 Gy × 3, but, interestingly, RT enhanced the efficacy of ICIs. The addition of COS did not further improve this treatment. However, the combination of ICIs and RT can result in severe side effects for patients [49]. COS are non-toxic and could therefore represent a safer treatment option for combination with ICIs. 

In an explorative study, 120 patients with lung cancer subjected to RT simultaneously received oral COS or a placebo. COS increased circulating lymphocytes in the blood, but the patient outcome was not assessed [50]. The addition of COS to chemotherapy in patients with resected pancreatic cancer was deemed safe in a phase II trial (ClinicalTrials.gov Identifier: NCT02767752), but no clinical benefit could be assessed due to a premature end of the trial [51]. Another recent study of chitosan in patients with prostate cancer (ClinicalTrials.gov identifier: NCT03712371) was recently terminated as the study was deemed unfavorable to continue due to unknown reasons. Studies of the treatment efficacy of COS combined with ICIs in patients with cancer are lacking. The results of the present study warrant further pre-clinical experiments to elucidate and conclude the role of COS as an anti-cancer drug and as an immunomodulatory agent that can enhance the efficacy of immunotherapy.

## Figures and Tables

**Table 1:** Immunohistochemical scoring of YKL-40 protein expression in syngeneic mouse tumors (n = 3 per group). Staining was assessed using a modified Allred scoring based on the sum of a proportion and an intensity score. The Allred scoring was converted to a scale from ‘−’ = no expression to ‘+’ = low expression, ‘++’ = moderate expression, and ‘+++’ = high expression.

Model	Tumor Expression	Stromal Expression
B16F10 melanoma	++	++
	++	++
	++	+
EO771.LMB breast	+++	+
	++	+
	++	+
LL2 lung	+	++
	+	++
	+	++
MC38 colon	++	++
	+	++
	+	++
PANO2 pancreatic	++	++
	+	+
	+	++

## Data Availability

Contact the corresponding author for data supporting the results of this study.

## Supplementary Materials

The following supporting information can be downloaded at: https://www.mdpi.com/article/10.3390/pharmaceutics14051046/s1, Figure S1: Gating strategy; Figure S2: Additional public scRNAseq data from patients with lung and colorectal cancer; Figure S3: Representative images of H&E stainings and immunostained YKL-40 protein expression in different syngeneic mouse cancer models; Figure S4: Cell composition in LL2 tumors treated with COS, ICI, or ICI + COS; Figure S5: Monotherapy with COS in the MC38 colon cancer model.
