# Supplementary material for: Chitooligosaccharides Improve the Efficacy of Checkpoint Inhibitors in a Mouse Model of Lung Cancer
Source: Pharmaceutics. 2022 May 12;14(5):1046. doi: 10.3390/pharmaceutics14051046 (PMC9147765; doi:10.3390/pharmaceutics14051046)
Supplement: Supplementary file 1 [file pharmaceutics-14-01046-s001.zip › pharmaceutics-1685840-supplementary.pdf]

Supplementary Materials

Supplementary Figure S1. Gating strategy.

Supplementary Figure S2. Additional public scRNAseq data from patients with lung and colorectal cancer.

Supplementary Figure S3. Representative images of H&E staining and immunostained YKL-40 protein expression in different syngeneic mouse cancer models.

Supplementary Figure S4. Cell composition in LL2 tumors treated with COS, ICI, or ICI+ COS.

Supplementary Figure S5. Monotherapy with COS in the MC38 colon cancer model.

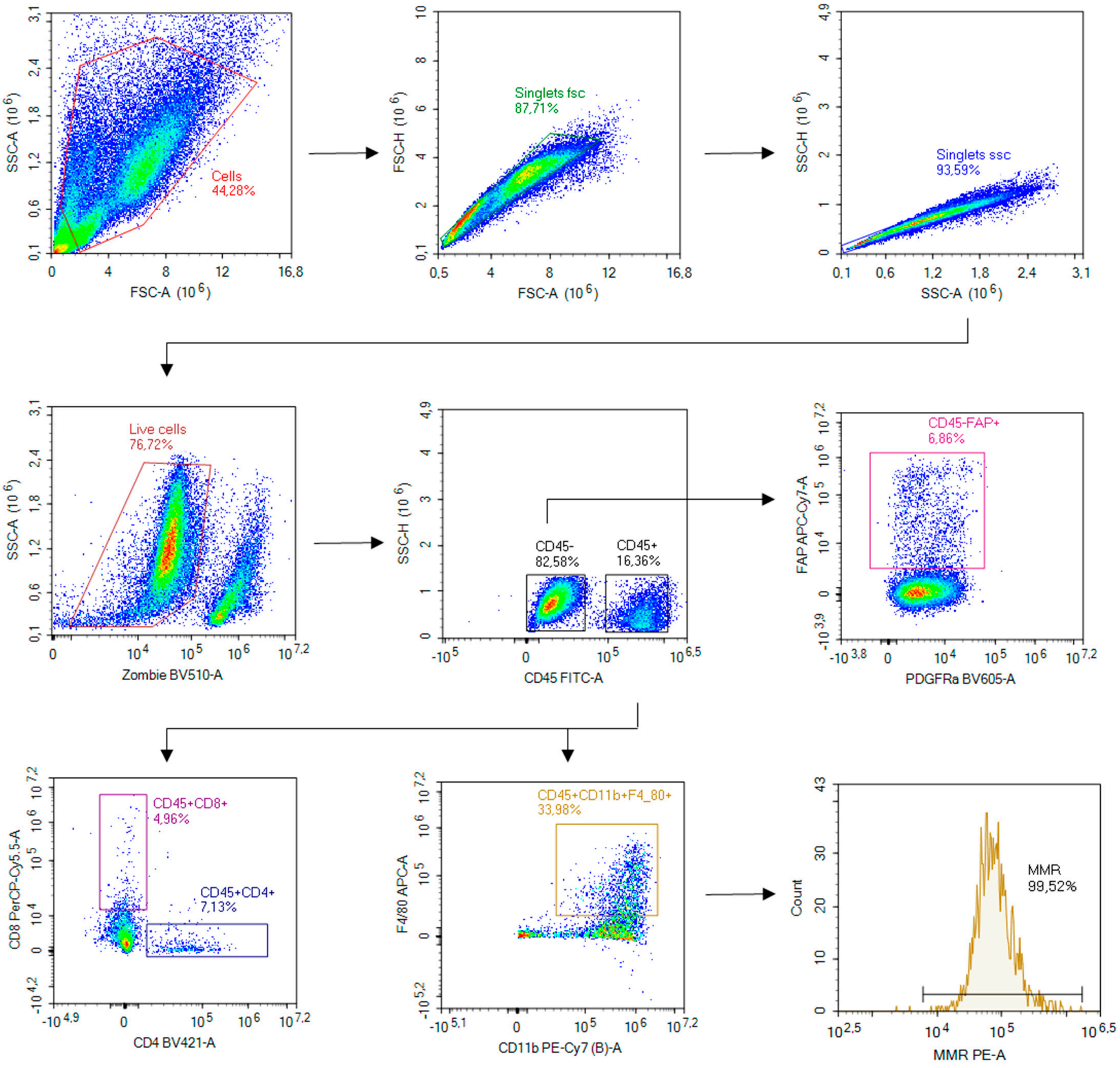

Supplementary Figure S1. Gating strategy for assessment of changes to cell populations in tumor digest from LL2 tumors (related to Supplemental Figure 3). Cells were initially gated as Cells → Singlets using forward scatter → Singlets using side scatter → CD45-/+. From CD45+ cell population, CD4+, CD8+ and CD11b+F4/80+ cells were gated. From CD11b+F4/80+, MMR expression was assessed. From the CD45- cell population, FAP+ were gated.

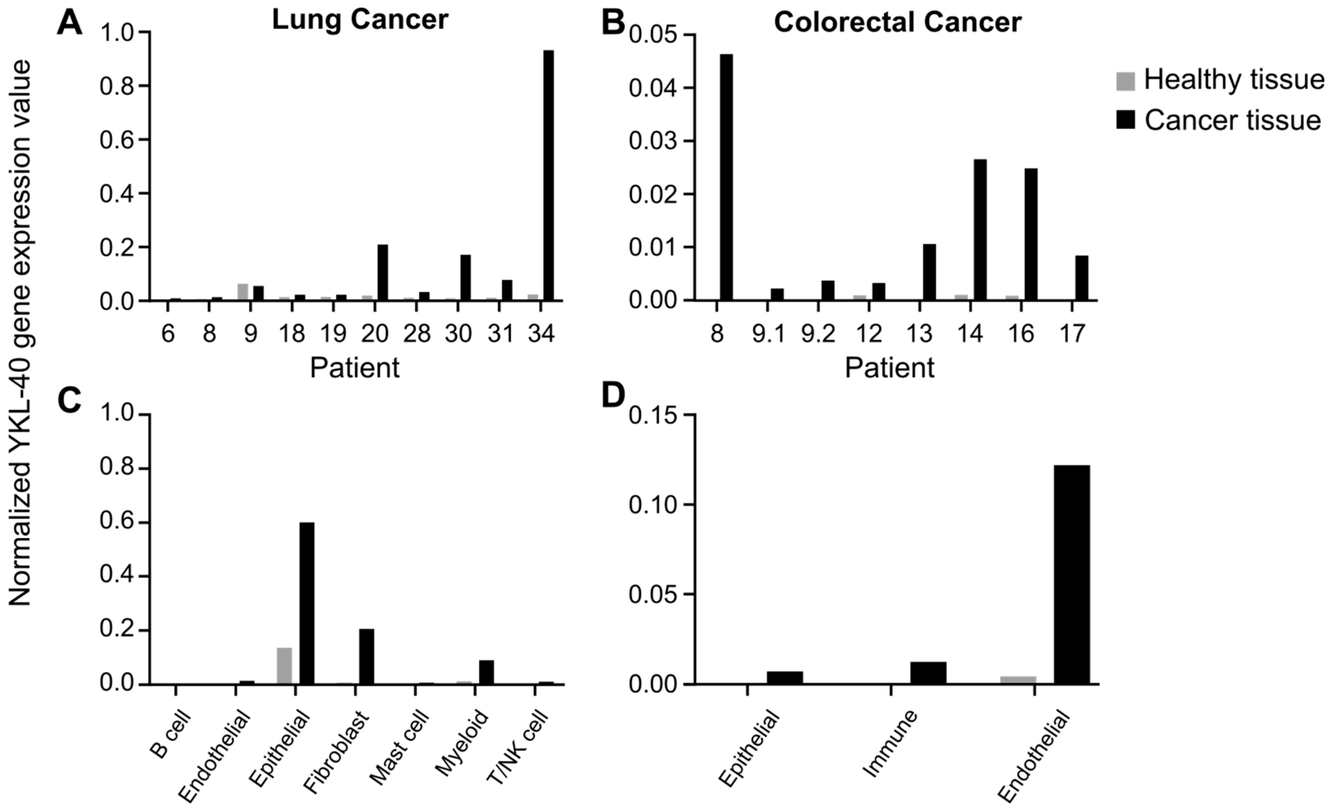

Supplementary Figure S2. Additional public scRNAseq data from patients with lung and colorectal cancer. Seurat-based normalized YKL-40 gene expression value comparing healthy tissue with cancer tissue in (**A, B**) individual patients and (**C, D**) cell subsets for: lung- (n = 10), GSE131907; and colorectal cancer (n = 8), GSE166555. For gene annotation of cell populations, refer to Kim et al. [26] (lung) and Uhlitz et al. [27] (colorectal).

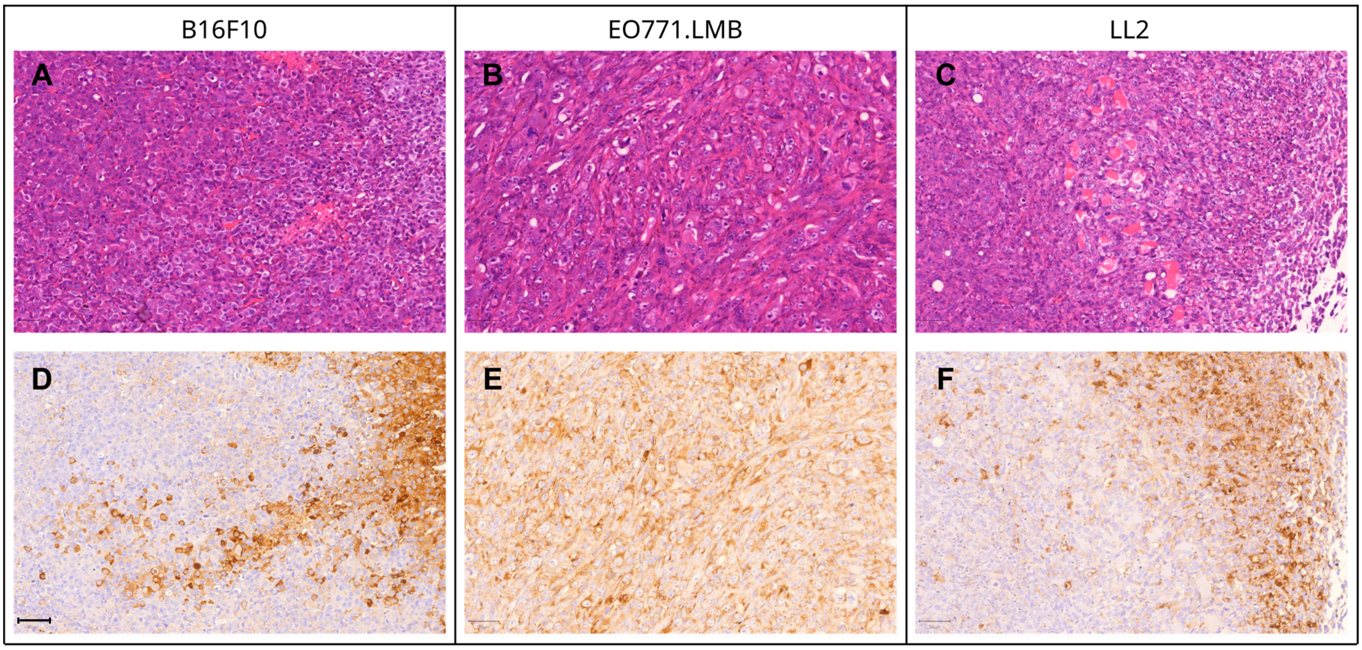

Supplementary Figure S3. Representative images of (**A-C**) H&E staining and (**D-F**) immunostained YKL-40 (brown) protein expression in different syngeneic mouse cancer models. YKL-40 is heterogeneously expressed, and large areas of the tumors are negative. The images are examples of (**D**) similar expression in cancer cells and stroma, (**E**) high expression in cancer cells and low in stroma, and (**F**) low expression in cancer cells and high in stroma. Scale bar 50 μm (all images).

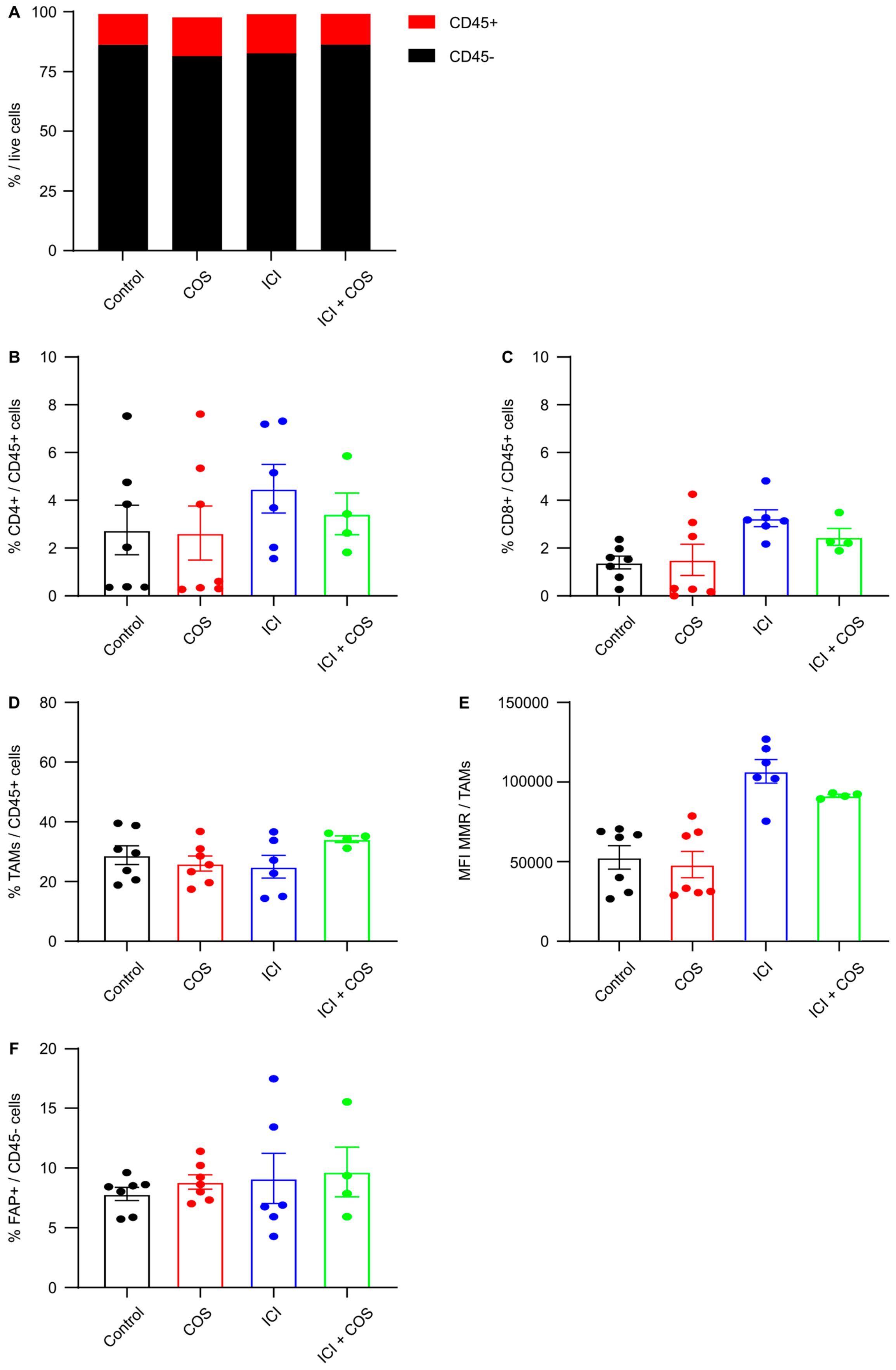

Supplementary Figure S4. Cell composition in LL2 tumors treated with COS, ICI, or ICI+ COS using multicolor flow cytometry (n = 4-7 per group). (**A**) CD45-/+ negative populations, illustrated as average of the treatment group. (**B-F)** Quantification of (**B**) CD4+ T cells, (**C**) CD8+ T cells, (**D**) TAMs, (**E**) MFI MMR on TAMs, and (**F**) FAP+ cells, mean ± SEM. TAMs: CD45+CD11b+F4/80+. Gating strategy is available in Supplementary Figure S1.

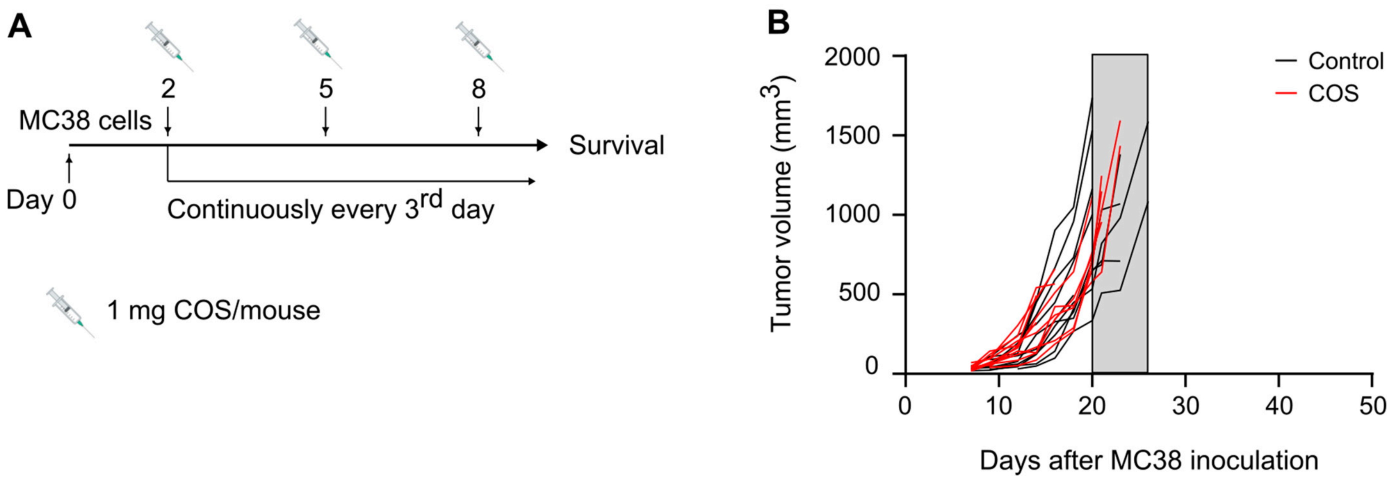

Supplementary Figure S5. Monotherapy with COS in the MC38 colon cancer model. (**A**) Treatment regimen and experimental setup of tumor study. (**B**) Individual tumor growth curves for C57BL/6 mice inoculated with $5*10^5$ MC38 cancer cells (n = 10 per group). Shaded areas included for easy comparison of the different groups represent the terminal tumor growth time frame of the control group. Abbreviation: COS: chitooligosaccharides.
